# Effects of a Physical Exercise Program on Physiological, Psychological, and Physical Function of Older Adults in Rural Areas

**DOI:** 10.3390/ijerph18168487

**Published:** 2021-08-11

**Authors:** Sunmi Kim, Eun-Jee Lee, Hyeon-Ok Kim

**Affiliations:** 1 College of Nursing, Jeonbuk National University, Jeonju 54896, Korea; eva0703@naver.com; 2 College of Nursing, Research Institute of Nursing Science, Jeonbuk National University, Jeonju 54896, Korea; khok@jbnu.ac.kr

**Keywords:** aged, walking, gymnastic, exercise, depression, cognitive

## Abstract

With the increase in the older population, there is a concern for health in older adults. This study aimed to develop a physical exercise program that combined walking and gymnastics for older adults residing in rural areas and to evaluate its effect on their physiological and psychological health and physical function. A quasi-experimental design was adopted. Participants were aged 65 years or older, with 94 and 130 participants in the experimental and control group, respectively. The program was implemented for seven months, from April–October 2016. Walking and gymnastics were performed once a week each, for about 60 and 50 min, respectively. Data were analyzed using the Chi-squared test, Fisher’s exact test, independent *t*-test, or Mann–Whitney U test. Results revealed that the experimental group participants displayed improved waist circumference (t = 1.70, *p* = 0.045), body mass index (U = 4691.00, *p* = 0.002), depressive symptoms (t = −2.94, *p* = 0.002), upper limb strength (t = 2.27, *p* = 0.012), and lower limb strength (t = 3.86, *p* < 0.001). Therefore, it can be presumed that the physical exercise program was effective and beneficial for older adults living in rural areas. This program is expected to contribute to maintaining and improving their health if implemented regularly in the future.

## 1. Introduction

Korea is entering an aging society, and the older Korean population is expected to continue to grow in the future [1]. In 2019, older adults over 65 years accounted for 14.9% of the total population in Korea, and this proportion has been predicted to reach 20.3% by 2025 [2].

With the increase in the older population, there is a simultaneous outburst of various health problems. In a healthcare service needs survey, 29.1% of respondents stated that they have three or more chronic health problems [3], and 39.7% of older adults responded negatively about their health status [4]. Moreover, the increasing older population also incurs higher healthcare costs. Older adults account for 33.3% of the Korea National Health Insurance costs [5]. Therefore, the hike in older adults’ medical expenses poses a grave threat to Korea’s medical finances. However, the existing healthcare services for older adults are insufficient. Furthermore, a Korean survey reported that over 50% of older adults do not receive adequate exercise and suffer from chronic diseases [6]. Therefore, a healthcare program for older adults is needed.

Exercise is known to be essential for ensuring the wellbeing of older adults. On the psychological front, exercise improves cognitive functions and depressive symptoms [7,8,9,10]. Aerobic exercise acts as a psychostimulant and facilitates cognitive processes, such as sensory and motor functioning [9]. Furthermore, group exercise programs enhance social interaction and mitigate worsening depression caused by social isolation [7]. Moreover, exercise provides physiological benefits by improving blood pressure and cholesterol, while decreasing the prevalence of cardiovascular diseases [11]. Additionally, it enhances the self-care ability of individuals [12,13,14].

Regular aerobic exercise is beneficial to individual health, regardless of age [14]. However, only 25% of older adults practice regular physical exercise [15]. According to the Centers for Disease Control and Prevention, physical activity is restricted by barriers such as a lack of time, social support, energy, motivation, skill, and facilities, as well as high costs, fear of injury, and poor weather conditions [16]. Therefore, accessibility of the program and social relationships are directly related to ensure continued exercise adherence [17]. Therefore, healthcare professionals need to consider these factors while developing physical activity interventions.

A safe exercise program must be developed for older adults. The American Cancer Society recommends four types of exercise for older adults: endurance, strength, balance, and stretching or flexibility exercises [15]. Walking is one such endurance exercise, and it is a safe aerobic exercise without straining joints or muscles [18]. Walking can also improve cardiovascular and limb strength, limb and trunk joint range, coordination, and balance [14]. Similarly, gymnastics can be categorized as a balance enhancement and a stretching or flexibility exercise [19]. These exercises can promote the performance of activities of daily living, improve posture, thwart the weakening of muscle fibers, and prevent falls [10,13,20].

Several existing studies have examined the effects of exercise. These studies implemented interventions combining aerobic exercise with education programs [8,13] and muscle-strengthening exercises [21], or they conducted the study with special populations (e.g., individuals with diabetes mellitus [12]). However, most exercise interventions could not be generalized due to the limited duration of testing, specific locations of testing, or applicability to restricted populations. Therefore, in this study, we aimed to develop an easy-to-administer exercise program for older adults for sustained implementation even after the completion of this research.

Thus, this study aimed to develop a physical exercise program that combined walking and gymnastics for older adults residing in rural areas and to evaluate the program’s effect on their physiological and psychological health and physical ability.

### Hypotheses of the Study

**Hypothesis** **1 (H1).** *Older adults who participate in the physical exercise program will depict improved physiological indicators (blood pressure, total cholesterol, weight circumference, and body mass index [BMI]) than those who do not.*

**Hypothesis** **2 (H2).** *Older adults who participate in the physical exercise program will indicate improved cognitive function and depressive symptoms than those who do not.*

**Hypothesis** **3 (H3).** *Older adults who participate in the physical exercise program will show improved physical ability compared to those who do not.*

## 2. Methods

### 2.1. Study Design

This study adopted a quasi-experimental design to investigate the effects of a physical exercise program on older adults’ physiological and psychological health and physical ability.

### 2.2. Participants

The inclusion criteria for experimental group participants specified individuals who:

(1) were aged 65 years or older,
(2) could walk independently,
(3) had an orientation to a person, time, and place,
(4) did not have any hearing problems that would hinder communication with others,
(5) attended more than 80% of the physical exercise program.

For control group participants, individuals who (1) were residing in different towns in the same county without physical exercise programs and (2) met experimental group participant inclusion criteria 1–4 were recruited to prevent treatment diffusion.

The sample size was calculated using the G*power 3.1.9.2 program. The effect size was determined based on a previous meta-analysis about the effect of exercise programs developed for older adults [22]. We performed a one-tailed independent *t*-test with the following parameters: medium effect size of 0.5, significance level of 0.05, and power of 0.8. The output indicated a minimum sample size of 51 for each of the two groups. In this study, we recruited 94 and 130 participants in the experimental and control group, respectively, thus fulfilling the sample size requirements.

### 2.3. Intervention

The physical exercise program comprised walking and performing gymnastics once a week each. Due to the cold climate in Korea from November–March, the exercise intervention in this study was conducted for a sufficient period of seven months from April–October.

The walking route was a 3.5 km flat course suitable for older adults within the participants’ residential village. In response to a previous survey, older adults suggested that they preferred walking around their residence [23]; thus, outdoor exercise was considered more effective than indoor exercise [24]. The community health center selected some villagers and provided them with an opportunity to train as walking specialists. These trained walking specialists, who lived in the same village, supervised the walking exercise. The walking exercise spanned over approximately 60 min and was performed in the following order: stretching for the warm-up exercise (5 min), walking along the designated path for the main exercise (50 min), and the cool-down exercise (5 min).

Gymnastics were performed within the village facility. Physical exercise specialists and community health practitioners accompanied the older adults to ensure safety during the program. The gymnastic exercise spanned over approximately 50 min. Community health practitioners informed participants regarding injury prevention and examined the participants’ condition before starting the gymnastic exercise. Gymnastic exercises were performed in the following order: whole body warm-up exercise (10 min); the main exercise comprised upper body, lower body, band, and dementia prevention exercises with vibrant music; and the cool-down exercise comprised full body stretching and acupressure.

### 2.4. Measures

Community health practitioners were educated on the measures to increase the assessment reliability. Moreover, face-to-face interviews were conducted and recorded by them, due to the participants’ old age.

#### 2.4.1. Physiological Variables

Height (cm), body weight (kg), waist circumference (cm), systolic blood pressure (SBP; mmHg), diastolic blood pressure (DBP; mmHg), and total cholesterol (mg/dl) were measured as physiological indicators. BMI was calculated by dividing body weight (kg) by height (m) squared.

#### 2.4.2. Psychological Variables

(1) Cognitive function

The Korean version of the Mini-Mental State Examination for Dementia Screening (MMSE-DS) [25,26], which evaluates Koreans without regular education, was used to evaluate cognitive function. The MMSE-DS comprises 19 items encompassing time orientation (year, season, date, day, month), place orientation (state, country, town, floor, place), immediate and delayed recall (three word [tree, car, cap] registration and recall), attention and calculation (Serial 7s), naming (watch, pencil), verbal repetition, three-stage command, visuospatial construction (copying interlocking pentagons), judgment, and abstract thinking.

The highest score on this scale is 30. Higher scores indicated better cognitive function. The tool reported high internal reliability in Kim’s study [25] and the Cronbach’s alpha was 0.82 in this study.

(2) Depression

In this study, depressive symptoms were measured using the Korean version of the Short-form Geriatric Depression Rating Scale (SGDS-K) [27], which comprises 15 items. Responses were scored as 1 (depressive) and 0 (non-depressive), and the total score was calculated. Higher scores indicated higher severity of depressive symptoms. Cronbach’s alpha was 0.89 in Cho’s [27] study and 0.84 in this study.

#### 2.4.3. Physical Ability

In this study, physical ability was measured by assessing bicep curls, back scratch, chair stand, one-leg standing with eye opening, and timed up and go.

(1) Upper limb strength (Bicep curls)

Upper limb strength was assessed using the bicep curls test [28]. Participants sat on a chair without armrests, with their back straight, and one arm was positioned to hold a dumbbell (male: 3 kg, female: 2 kg). Upon hearing the start signal, participants lifted the dumbbell above their elbow and lowered it; this process continued for 30 s, and the staff counted the frequency of lifting the dumbbell within 30 s. Higher frequency indicated better upper limb strength.

(2) Upper body flexibility (Back scratch)

The back scratch test was performed to assess upper body flexibility [28]. Participants positioned any one hand over the same-side shoulder and were asked to lower it as far down as possible with their palm facing the back. Further, the other hand was directed behind the back through the waist on the same side, and they were asked to raise it as far as possible with the palm facing outward. Participants were asked to try and touch the middle fingers of both hands together as much as possible, while a staff member measured the distance (cm) between these middle fingers. Depending upon whether the middle fingers overlapped or did not touch, a positive or negative score was assigned, respectively. Thus, higher scores indicated better upper limb flexibility.

(3) Lower limb strength (Chair stand)

We performed a chair stand test to assess lower limb strength [28]. Participants comfortably sat on a chair without armrests and crossed both arms over their chest. A staff member recorded the number of times participants completely got up from the chair and sat back. Higher frequency indicated better lower limb strength.

(4) Balance (One-leg standing with eye opening)

Balance was assessed using the one-leg standing test with open eyes [29]. Participants were asked to stand with their eyes open, then lift one foot, and stay still for as long as possible. A staff member recorded the time taken to place their foot back on the floor. Time recording was stopped when participants moved their arms or feet to grab an object or to maintain their balance. Higher recorded time indicated better balance.

(5) Mobility (Timed up and go)

The timed up and go test was performed to assess mobility [30]. Participants began the test by sitting on a chair. A staff member recorded the time taken by the participant to get up from the chair, walk along the line drawn on the floor, and return to sit back in their own chair. Lower recorded time indicated better mobility.

#### 2.4.4. General Characteristics

Participants provided information about age, sex, education level, economic status, and marital status.

### 2.5. Statistical Analysis

Data were analyzed using SPSS Version 26.0 (SPSS INC., Chicago, IL, USA). Participants’ general characteristics were analyzed using descriptive statistics. Normality was confirmed through skewness and kurtosis tests.

Chi-squared test, Fisher’s exact test, independent *t*-test, Mann–Whitney U test, or ANCOVA was performed to compare the outcomes between the experimental and the control group participants. All analyses were performed as one-tailed tests with statistical significance set at *p* < 0.05.

## 3. Results

### 3.1. Homogeneity Test of Participants

Except for upper body flexibility (t = −2.01, *p* = 0.046), none of the participant characteristics reported significant differences between the two groups (Table 1).

### 3.2. The Effects of the Physical Exercise Program

Table 2 presents the effect of the physical exercise program on physiological indicators. The experimental and control group participants reported significant differences in waist circumference (t = −1.70, *p* = 0.045) and BMI (U = 4691.00, *p* = 0.002) before and after the exercise program. However, SBP, DBP, and total cholesterol (U = 6063.50, *p* = 0.461) did not report any significant differences between the experimental and the control group.

Table 3 presents the effects of the physical exercise program on cognitive function and depression. A significant difference was observed in depression (t = −2.94, *p* = 0.002) before and after exercise between the experimental and control group. However, there was no significant difference in cognitive function.

Table 4 presents the effects of the physical exercise program on the physical ability. There was a significant difference between the experimental and control group in the upper limb strength (t = 2.27, *p* = 0.012) and lower limb strength (t = 3.86, *p* < 0.001) before and after the exercise program. However, there was no significant difference in balance and mobility between the two groups.

## 4. Discussion

This study aimed to determine the effects of a physical exercise program on the physiological indicators, cognitive function, depression, and physical ability of older adults residing in rural areas.

In this study, the physical exercise program was effective at reducing waist circumference and BMI; however, there was no effect on blood pressure or total cholesterol. This finding is consistent with a secondary analysis conducted using the national insurance data [31] and with the study results of older adults with diabetes [8]. The National Institute of Aging [32] recommended the following lifestyle changes to prevent hypertension: maintain a healthy weight, exercise regularly, consume a healthy diet, cut down on salt intake, reduce alcohol consumption, quit smoking, improve sleep schedule, and manage stress. In addition, the following were risk factors for high cholesterol: smoking, being overweight, fat- and sugar-rich diet, and sedentary lifestyle [33]. Although the exercise program for older adults in this study did not show an effect on blood pressure and cholesterol, it was effective in weight control. Therefore, it is essential to develop more integrated interventions that combine exercise, diet, education, and group activities, including physical exercise, to enable improvements in blood pressure and cholesterol. Additionally, older adults are likely to have one or more chronic diseases. Thus, it is necessary to compare the differences between the experimental and control group participants, after controlling for these diseases, to highlight the effect of exercise on blood pressure and total cholesterol.

The physical exercise program in this study was effective at lowering the level of depression. This finding is consistent with a previous study [34]. Therefore, physical exercise is important for older adults’ mental health [35]. The major factors influencing depression are sex (female), somatic illness, cognitive and functional impairment, lack or loss of social contact, and history of depression [36,37]. Therefore, a more integrated intervention program is required to reduce depression in older adults, and we recommend incorporating physical exercise into the intervention. Additionally, in this study, the increase in the depression scores of the control group older adults could be due to seasonal changes or environmental factors amidst the intervention period.

The cognitive function level of all participants in this study was consistent with a previous study using the same instrument [38]. Moreover, in line with another existing study, the physical exercise program in this study had no effect on cognitive function [39]. However, Park and Sohng [7] reported an improvement in cognitive function in their study after implementing an integrated program encompassing chronic disease management education, safety education, art therapy, and physical exercise. Therefore, we recommend a complex intervention program for cognitive function improvement.

The current physical exercise program was effective at improving lower limb strength and upper arm strength of older adults. An existing study reported the effectiveness of walking for improving lower limb muscle endurance and full body endurance among older adults [24]. Based on these results, future researchers must incorporate aerobic exercise as well as gymnastics to improve muscle strength and flexibility while developing an exercise program for older adults.

Participants in both groups of this study mostly comprised older female adults. A previous study reported that females were more motivated by social factors than males [40]. In addition, there were sex differences in the preferences for the type of exercise. Females preferred scheduled activities, while males preferred vigorous skill-based activities that required practice and involved competition [40]. Accordingly, the lower male participant rate in this study may have occurred due to the reduced attractiveness of the group physical exercise program provided to the older adults in this study. Therefore, these factors should be considered while developing a program to increase the participation rate of the male older adults.

The limitations of this study are as follows. First, this study used a convenience sample in one area, therefore findings may not be generalized to all older adults. The program encompassed two types of exercises only: walking and gymnastics. Second, this study was conducted in one rural area only, and thus its findings may not be generalizable for all older adults.

However, the current physical exercise program was developed for older adults in rural areas to be easily applied alongside their daily life, and it was effective in promoting health. Healthcare costs can be saved by older adults attending regular exercise programs. Furthermore, free or inexpensive access to fitness facilities and programs can motivate older adults to exercise [41]. Additionally, some trained villagers supervised the walking exercise in this study. Such supervisors aided in preventing injuries among participants during the exercise throughout the intervention period. Fewer injuries incur lower medical expenses and enable older adults to engage in regular exercise [41]. South Korea is one of the world’s fastest aging societies [42]. Thus, regular physical exercise provision can promote physical and psychological health for older adults. Moreover, older adults with health support receive the opportunity to participate in various activities, which results in the development of various industries for the elderly, such as the silver economy and tourism [43].

Based on the current study’s findings, we propose the following future research recommendations. First, researchers should develop more integrated intervention programs accounting for gender differences, which can be implemented regularly among older adults in rural areas. Second, the current exercise program did not demonstrate any significant effects on blood pressure, cholesterol, or cognitive function; however, future studies must investigate the long-term effects of exercise through follow-up. Third, this study did not employ participants’ comorbidities data. Future researchers should consider investigating this and comparing its differences between the two groups. Fourth, we evaluated the abstract concepts of depression and cognitive function using widely used instruments with high reliability and validity; however, there may be gaps between the measured values and older adults’ actual experience of depression and cognitive function. Therefore, we suggest integrating various alternative measures in future research to avoid these gaps. Lastly, participants did not undergo the exercise intervention during winter, due to reduced immunity and increased morbidity and mortality caused by outdoor exercise in winter [44,45]. Therefore, it is necessary to develop safe winter-based exercise interventions for older adults.

## 5. Conclusions

This study developed and implemented an easy-to-administer physical exercise program for older adults over seven months and investigated its effects. The program improved depressive symptoms and physical function. Therefore, the physical exercise program was effective and beneficial for older adults living in rural areas, and it is expected to contribute to maintaining and improving their health, if it is regularly implemented in the future.

## Figures and Tables

**Table 1:** Homogeneity test for general characteristics and dependent variables at baseline between two groups.

Variables		Experimental Group (n = 94)	Control Group (n = 130)	*t* or x^2^	*p*
		n (%) or Mean ± SD	n (%) or Mean ± SD		
Age		75.37 ± 5.89	74.72 ± 5.68	0.83	0.407
Sex	Male	5 (5.3)	15 (11.5)	2.60	0.154
	Female	89 (94.7)	115 (88.5)		
Education Level	None (cannot read-write)	18 (19.1)	29 (22.3)	1.62 ^†^	0.915
	None (read only)	26 (27.7)	31 (23.8)		
	None (can read-write)	17 (18.1)	27 (20.8)		
	Elementary school	25 (26.6)	34 (26.2)		
	Middle school	3 (3.2)	5 (3.8)		
	High school	5 (5.3)	4 (3.1)		
Economic status	Affordable	11 (11.7)	20 (15.4)	4.86	0.090
	Moderate	70 (74.5)	79 (60.8)		
	Insufficient	13 (13.8)	31 (23.8)		
Income level	Basic livelihood security recipient	1 (1.1)	7 (5.4)	4.09 ^†^	0.125
	Next lowest income bracket	16 (17.0)	29 (22.3)		
	General income earner	77 (81.9)	94 (72.3)		
Marital status	Married	46 (48.9)	62 (47.7)	2.40 ^†^	0.531
	Divorced/Separated	1 (1.1)	0 (0.0)		
	Bereaved	47 (50.0)	66 (50.8)		
	Other	0 (0.0)	2 (1.5)		
Waist circumference (cm)		89.21 ± 8.96	87.70 ± 8.56	1.27	0.204
Systolic blood pressure (mmHg)		133.68 ± 16.28	131.80 ± 17.50	0.82	0.415
Diastolic blood pressure (mmHg)		78.34 ± 10.74	77.38 ± 9.19	0.72	0.475
Total cholesterol (mg/dl)		184.79 ± 37.46	194.63 ± 42.30	−1.80	0.073
Body Mass Index (kg/m^2^)		24.74 ± 3.44	24.65 ± 3.38	0.19	0.853
Cognitive function		23.22 ± 3.94	23.61 ± 4.13	−0.70	0.484
Depression		3.02 ± 2.87	3.58 ± 3.64	−1.29	0.197
Upper limb strength (frequency)		16.13 ± 4.70	16.51 ± 5.00	−0.58	0.565
Upper body flexibility (cm)		−16.69 ± 12.27	−12.63 ± 18.06	−2.01	0.046
Lower limb strength (frequency)		12.09 ± 3.41	11.51 ± 4.10	1.15	0.266
Balance (sec)		15.65 ± 22.69	14.12 ± 20.90	0.52	0.604
Mobility (sec)		11.03 ± 4.21	11.81 ± 5.33	−1.17	0.241

^†^ Fisher’s exact test; SD = standard deviation.

**Table 2:** Comparing the effects of the physical exercise program on physiological indicators.

Variables	Group	Pretest Mean ± SD	Posttest Mean ± SD	Differences Mean ± SD	*t*^†^ or U	*p* ^‡^
Waist circumference (cm)	Exp	89.21 ± 8.96	87.90 ± 9.00	−1.31 ± 3.72	−1.70	0.045
	Con	87.70 ± 8.56	87.40 ± 8.24	−0.30 ± 4.84		
Systolic blood pressure (mmHg)	Exp	133.68 ± 16.28	130.33 ± 15.00	−3.35 ± 11.76	5607.50 ^§^	0.147
	Con	131.80 ± 17.50	130.81 ± 15.16	−0.99 ± 16.03		
Diastolic blood pressure (mmHg)	Exp	78.34 ± 10.74	77.22 ± 9.17	−1.12 ± 9.38	−0.73	0.234
	Con	77.38 ± 9.19	77.15 ± 8.97	−0.23 ± 8.72		
Total cholesterol (mg/dL)	Exp	184.79 ± 37.46	176.78 ± 34.49	−8.01 ± 41.09	6063.50 ^§^	0.461
	Con	194.63 ± 42.30	182.47 ± 48.73	−12.16 ± 46.68		
Body Mass Index (kg/m^2^)	Exp	24.74 ± 3.44	24.22 ± 3.19	−0.52 ± 1.44	4691.00 ^§^	0.002
	Con	24.65 ± 3.38	24.49 ± 2.88	−0.16 ± 1.76		

^†^ independent *t*-test; ^‡^ one-tailed *p* value; ^§^ Mann–Whitney U test; Exp = experimental group; Con = control group; SD = standard deviation of the mean.

**Table 3:** Comparing the effects of the physical exercise program on cognitive function and depression.

Variables	Group	Pretest Mean ± SD	Posttest Mean ± SD	Differences Mean ± SD	*t*^†^ or U	*p* ^‡^
Cognitive condition	Exp	23.22 ± 3.94	23.38 ± 3.75	0.16 ± 1.55	5706.00 ^§^	0.173
	Con	23.61 ± 4.13	23.83 ± 4.29	0.22 ± 2.16		
Depressive symptom	Exp	3.02 ± 2.87	2.69 ± 2.58	−0.33 ± 2.52	−2.94	0.002
	Con	3.58 ± 3.64	4.38 ± 3.61	0.80 ± 3.05		

^†^ independent *t*-test; ^‡^ one-tailed *p* value; ^§^ Mann–Whitney U test; Exp = experimental group; Con = control group; SD = standard deviation of the mean.

**Table 4:** Comparing the effects of the physical exercise program on physical ability.

Variables	Group	Pretest Mean ± SD	Posttest Mean ± SD	Differences Mean ± SD	*t*^†^ or U	*p* ^‡^
Upper limb strength (frequency)	Exp	16.13 ± 4.70	18.07 ± 5.16	1.95 ± 3.42	2.27	0.012
	Con	16.51 ± 5.00	17.19 ± 6.06	0.68 ± 4.53		
Upper body flexibility (cm)	Exp	−16.69 ± 12.27	−16.77 ± 13.63	−0.07 ± 10.82	1.74 *	0.189
	Con	−12.63 ± 18.06	−16.31 ± 14.89	−3.69 ± 13.80		
Lower limb strength (frequency)	Exp	12.09 ± 3.41	13.21 ± 3.70	1.13 ± 3.22	3.86	<0.001
	Con	11.51 ± 4.10	11.11 ± 4.04	−0.40 ± 2.69		
Balance (sec)	Exp	15.65 ± 22.69	26.06 ± 31.66	10.42 ± 30.98	−0.45	0.327
	Con	14.12 ± 20.90	26.34 ± 31.56	12.22 ± 28.79		
Mobility (sec)	Exp	11.03 ± 4.21	11.46 ± 3.46	0.43 ± 3.23	5646.50 ^§^	0.166
	Con	11.81 ± 5.33	13.53 ± 11.10	1.73 ± 10.73		

^†^ independent *t*-test; ^‡^ one-tailed *p* value; * ANCOVA; ^§^ Mann–Whitney U test; Exp = experimental group; Con = control group; SD = standard deviation of the mean.

## Data Availability

The data used and/or analyzed during the current study are available from the corresponding author upon reasonable request.
